# Serum and Cerebrospinal Fluid Autoantibodies in Patients with Neuropsychiatric Lupus Erythematosus. Implications for Diagnosis and Pathogenesis

**DOI:** 10.1371/journal.pone.0003347

**Published:** 2008-10-06

**Authors:** Hilda Fragoso-Loyo, Javier Cabiedes, Alejandro Orozco-Narváez, Luis Dávila-Maldonado, Yemil Atisha-Fregoso, Betty Diamond, Luis Llorente, Jorge Sánchez-Guerrero

**Affiliations:** 1 Department of Immunology and Rheumatology, Instituto Nacional de Ciencias Médicas y Nutrición Salvador Zubirán, México, Distrito Federal, México; 2 Department of Neurology, Instituto Nacional de Ciencias Médicas y Nutrición Salvador Zubirán, México, Distrito Federal, México; 3 The Feinstein Institute for Medical Research, Manhasset, New York, United States of America; Centre de Recherche Public-Santé, Luxembourg

## Abstract

**Background:**

Despite the uncertainty in the diagnosis of neuropsychiatric involvement in systemic lupus erythematosus (SLE), attempts have been made to record the association of certain antibodies in serum with neuropsychiatric (NP) manifestations. We aimed to assess the behaviour and the association of serum and cerebrospinal fluid (CSF) autoantibodies with NP manifestations in SLE patients (NPSLE).

**Methodology/Principal Findings:**

Forty-seven SLE patients, hospitalized because of NP manifestations were included. They were evaluated at hospitalization and six months later, and serum and CSF samples were obtained at each evaluation. As controls, serum samples were taken from 49 non-NPSLE patients at hospitalization and six months later; serum and CSF samples were also obtained from 6 SLE patients with septic meningitis, 16 surgical SLE patients and 25 patients without autoimmune diseases. Antinuclear, anti-dsDNA, anti-ribosomal P, Anti-N-Methyl-D-Aspartate receptor (NMDAR), anti-cardiolipin, and anti-β2 glycoprotein-I antibodies were measured. In serum, anti-ribosomal P, anti-NMDAR, and other antibodies did not differentiate among SLE groups, and the levels of all antibodies were similar among the SLE groups. Six-months later, this scenario remained unchanged and the decrease in the levels of some autoantibodies reflected a decline in disease activity, rather than a change in NPSLE. In CSF, only the presence and the levels of anti-NMDAR antibodies showed a characteristic distribution in central NPSLE and septic meningitis patients. Six months later the prevalence of most antibodies in CSF did not change, however the levels of anti-dsDNA, anti-ribosomal P, and anti-NMDAR decreased.

**Conclusion:**

In NPSLE, autoantibodies in serum do not reflect their behaviour in CSF. All autoantibodies were elevated in septic meningitis reflecting the global penetration of serum antibodies into the CSF in this condition. Anti-NMDAR antibodies in CSF identified patients with central NPSLE; their continued presence in CSF 6 months after neurologic symptoms raise questions regarding the conditions under which they are pathogenic.

## Introduction

Systemic lupus erythematosus (SLE) is an autoimmune disease characterized by distinctive tissue pathology. Despite the presence of autoantibodies and tissue damage, the relationship between them remains controversial and clear explanations for many of the clinical features are yet to be given [1].

Central nervous system (CNS) involvement is a commonly encountered situation in which diagnostic certainty is lacking [2]. The clinical manifestations are diverse, ranging from mild affective disorders to seizures, cognitive dysfunction and stroke. Other conditions capable of causing neuropsychiatric disorders such as severe hypertension and corticosteroid therapy frequently coexist [3]. Furthermore, no laboratory or radiographic tests have been reported that are both sensitive and specific in establishing the diagnosis of NPSLE. In spite of this, attempts have been made to record the association of certain antibodies, e.g., anti-ribosomal P, anti-NMDAR, anti-phospholipids, with NPSLE, since the former usually accompany the latter. Some reports have assessed the role of these antibodies in the diagnostic evaluation of NPSLE [4]–[8] and others have involved them in the pathogenesis of NP manifestations [9]–[17]. Nonetheless, the question that remains unanswered is whether these antibodies are a consequence of NPSLE or they are one of its causes. A third option is that they are merely an epiphenomenon.

The aim of the present study was to assess the association of serum and CSF autoantibodies with NP manifestations in SLE patients, and to provide insight into whether they participate in the pathogenesis of NPSLE. According to the results observed, serum autoantibodies may be misleading as a diagnostic tool in NPSLE, while in CSF, their presence in SLE patients with septic meningitis and central NPSLE in remission raise questions regarding the circumstances in which they may be pathogenic.

## Methods

### Objective

To assess the behaviour and the association of serum and CSF autoantibodies with NP manifestations in NPSLE patients.

### Participants

Forty-seven SLE patients, [American College of Rheumatology (ACR) criteria [18], hospitalized between February 2003 and June 2005, because of NP manifestations were included. All patients were evaluated by the study rheumatologists and neurologists, at hospitalization and six months later using a standardized protocol, including disease activity assessment using the Systemic Lupus Erythematosus Disease Activity Index 2000 (SLEDAI-2K) [19]. At hospitalization, information on socio-demographic data, SLE characteristics (i.e. age at diagnosis defined as the date of the fourth lupus criteria, disease duration, SLE criteria accumulated, etc.), and treatment was gathered, and the medical records were reviewed to collect additional information, including chronic damage accrual using the Systemic Lupus International Collaborating Clinics/ ACR Damage Index [20]. A serum sample was obtained in all the patients at hospitalization and in 39 patients six months later. A CSF sample was obtained, in 40 patients at hospitalization and in 30 patients, who consented a lumbar control punction, six months later.

Neuropsychiatric manifestations were classified using the ACR nomenclature for neuropsychiatric lupus syndromes [21], and the patients were categorized in a central NPSLE group: seizure disorders 16, severe refractory headache 9, acute confusional state 8, cerebrovascular disease 7, psychosis 1, and pseudotumor cerebri 1; and a peripheral NPSLE group: multiplex mononeuritis 3, transverse myelitis 1, and polyneuropathy 1. Neurpsychiatric manifestations were attributed to SLE given that there were no exclusion factors for it [21], and none of the patients had any of the minor NP events which have been reported with a comparable frequency in the general population [22]

As controls, 49 hospitalized SLE patients with no history of NP manifestations, malignancies, or severe infections (Non-NPSLE group), matched by age (±5 years) and gender to the NPSLE patients were studied. They were also evaluated by the study rheumatologists at hospitalization and six months later identical to the NPSLE patients. A serum sample was obtained in all the patients at hospitalization and in 40 patients six months later. Reasons for hospitalization were: lupus disease activity 43 (SLE diagnosis, renal, hematological, neumonitis, serositis, hepatitis, fever), pulmonary thromboembolism 2, and miscellaneous 4.

A serum sample was also obtained from 6 SLE patients with septic meningitis (SLE septic meningitis group), 16 SLE patients without NP manifestations ever who underwent an elective surgery (SLE surgical group), and 25 patients with neither autoimmune diseases nor NP manifestations (Non-autoimmune group) who also underwent elective surgery. A CSF sample was obtained from 5 patients of the septic meningitis group, 16 of the surgical group, and in 17 of the non-autoimmune group. Patients in the latter two groups gave written permission to donate the CSF sample during the spinal block.

In all the cases, serum and CSF samples were obtained during the clinical assessment upon the arrival of the patients at the hospital. Serum was collected, and CSF was centrifuged at 12,000 g. Serum and the CSF supernatant were immediately frozen (<30 minutes) at −86°C until assayed for the presence of autoantibodies.

### Detection of autoantibodies

Anti-dsDNA, anti-cardiolipin, and anti-β2 glycoprotein I antibodies of the IgG isotype were detected by immunoenzymatic assay (EIA) according to the manufacturer's recommendations (The Binding Site, Birmingham, UK). Similarly, anti-ribosomal P antibodies of the IgG isotype were detected by EIA (Orgentec Diagnostika, Germany). In all instances serum samples were diluted 1∶100 and CSF samples were tested undiluted. Anti-NMDAR antibodies of the IgG isotype were detected by ELISA as previously described [17]. Briefly, DWEYS peptide was adsorbed to microtiter plates in 0.1M NaHCO_3_ (pH 8.6) overnight at 4°C. Serum was assayed at 1∶50 and 1∶100 dilution and developed with an enzyme-tagged antibody to human IgG. Antinuclear antibodies of the IgG isotype were detected by indirect immunofluorescence according to the manufacturer's recommendations (The Binding Site). Serum samples were diluted 1∶40 and CSF samples were tested undiluted. Three experts read all samples and results were discussed and registered by consensus. The sera cut-off values for all evaluated autoantibodies were set above the percentile 90^th^ of a separate control group of 100 healthy individuals (ANA >1∶40, anti-dsDNA <9.6 IU/mL, anti-ribosomal P<10.0 U/mL, anti-cardiolipin <11.5 UGPL, anti-β2 glycoprotein I <2.5 U/mL, anti-NMDAR 19.0 O.D.), and the CSF cut-off values for all evaluated autoantibodies were set above the 90^th^ percentile of the 17 patients with non-autoimmune diseases (anti-dsDNA ≤9.62 IU/mL, anti-ribosomal P≤9.96 U/mL, anti-cardiolipin ≤4.5 UGPL, anti-β2 glycoprotein I ≤2.5 U/mL, anti-NMDAR ≤40.0 O.D.). All autoantibodies were detected blinded to the status of the patients.

### Ethics

The study was approved by the Institutional Committee of Biomedical Research and all patients signed an informed consent.

### Statistical methods

Categorical variables were compared using chi-squared or Fisher's exact test, and continuous variables using Student's t-test, Mann-Withney U test, Wilcoxon signed-ranks test, paired t-test, or one-way ANOVA. It was planned in advance to analyze the prevalence and the levels of the autoantibodies studied between the Central NPSLE and each one of the other SLE groups, so corrections for the number of comparisons were not considered. P value was set at <0.05, two-tailed. Analysis was performed using the SPSS 12.0 computer program.

## Results

### Population characteristics

At entry, the mean±SD age of the NPSLE central and peripheral patients was 31.5±11.6 and 23.8±6.1 years, respectively; no difference in age was observed across the patient study groups (P = 0.22). As compared with the NPSLE group, non-NPSLE and SLE-surgical patients had longer disease duration and the former group also required immunosuppressants more often. All SLE groups, except the surgical group, had moderate/severe disease activity and were taking prednisone at high doses. Other demographic and clinical characteristics were similar among the study groups (Table 1).

**Table 1:** Demographic and clinical characteristics of the study patients at hospitalization.

	Central NPSLE (n = 42)	Peripheral NPSLE (n = 5)	Non-NP SLE (n = 49)	SLE-Surgical (n = 16)	SLE-septic meningitis (n = 6)	Non-autoimmune (n = 25)
Age, *years* A	31.5±11.6	23.8±6.1	30.7±12.0	37.8±9.8	29.7±10.3	37.5±15.3
Male/female	7/35	0/5	4/45	2/14	0/6	6/19
SLE duration, *years*	3.9±4.4	1.8±2.4	8.8±6.5^B^	8.8±7.2^C^	3.2±3.8	–
SLE criteria, *No.*	5.4±2.1	6±3.2	5.0±2.4	6.1±2.0	5.3±1.5	–
SLEDAI–2K score, at baseline	14.9±9.4	13.2±8.7	11.5±7.9	3.8±1.5^B^	10.6±6.0	–
SLEDAI–2K score, at 6 months	5.4±5.8	4.0±6.9	5.9±6.6	–	–	–
SLICC/ACR DI score	0.7±1.2	0.4±0.9	0.4±0.8	0.8±0.4	0.3±0.5	–
Prednisone use, *%*	91	100	92	6.3^B^	100	–
Prednisone dose, *mg/day*	44.7±24.3	52±13.0	41.7±22.5	–	55±12.2	–
Immunosuppressants use, *%*	48	100	71^D^	25	17^B^	–

A Except where indicated otherwise, values are the mean±SD. SLE = systemic lupus erythematosus; NPSLE = neuropsychiatric SLE; SLEDAI-2K = Systemic Lupus Erythematosus Disease Activity Index 2000 update; SLICC/ACR DI = Systemic Lupus International Collaborating Clinics/ American College of Rheumatology Damage Index.

B P<0.001 versus Central NPSLE

C P = 0.003 versus Central NPSLE

D P = 0.02 versus Central NPSLE

SLE was considered the cause of the NP events in all the patients; in 21 (45%) patients, no associated factors for the NP manifestations were identified, and in 26 (55%) patients, concurrent, non-exclusion factors, i.e. metabolic abnormalities, high doses of steroids, arterial hypertension were identified [21].

Non-autoimmune disease patients underwent elective surgery because of: bone marrow donors (7), hysterectomy (8), Tenchkoff catheter (3), lower limb amputation (2), saphenectomy (2), hydrocele (1), circumcision (1), and inguinal hernioplasty (1). Septic meningitis was due to: *Streptococcus pneumoniae* (2), *Lysteria monocytogenes* (1), *Cryptococcus neoformans* (1), *Mycobacterium tuberculosis* (1), and *Staphylococcus sp*. (1).

### Autoantibodies in serum

The prevalence of all the autoantibodies was higher across all the SLE groups than in patients without autoimmune disease.

Anti-ribosomal P antibodies were detected in most patients with central and peripheral NPSLE, but also in most patients with septic meningitis. Moreover a third of patients in the non-NPSLE and the surgical groups were positive for these antibodies.

Anti-NMDAR antibodies were detected in all the SLE patient groups, except in those with peripheral NPSLE; although the prevalence was higher in the central NPSLE, non-NPSLE and septic meningitis groups than in surgical patients, this difference was not statistically significant. The prevalence of all the other antibodies did not show clear differences across the SLE groups, and the levels of all antibodies did not differ among the SLE groups (Table 2).

**Table 2:** Prevalence and levels of autoantibodies in serum from NPSLE patients and controls.

Autoantibodies positive, no. (%)	Central NPSLE (42)	Peripheral NPSLE (n = 5)	Non-NP-SLE (n = 49)	SLE-Surgical (n = 16)	SLE-septic meningitis (n = 6)	Non-autoimmune (n = 25)
Antinuclear	33 (79)	4 (80)	44 (90)	14 (88)	6 (100)	3 (12)^B^
Anti-ds DNA	34 (81)	5 (100)	37 (76)	11 (69)	4 (67)	4 (16)^B^
Anti-ribosomal P	25 (60)	4 (80)	15 (31)^C^	6 (38)	5 (83)	3 (12)^B^
Anti-cardiolipin (IgG)	8 (19)	2 (40)	13 (27)	2 (13)	2 (33)	1 (4)
Anti-B2-Glycoprotein I (IgG)	26 (62)	4 (80)	27 (55)	9 (56)	5 (83)	3 (12)^C^
Anti-N-Methyl-D-Aspartate receptor^A^	11 (32)	0 (0)	13 (27)	2 (17)	2 (40)	2 (8)^D^
**Autoantibodies levels** E						
Anti-ds DNA	369.5±658.6	163.9±292.5	523.9±723.6	184.3±530.8	427.8±506.4	10.6±0.66
Anti-ribosomal P	94.9±153.3	106.9±188.7	96.7±117.6	126.3±184.4	148.5±196.3	16.4±3.8
Anti-cardiolipin (IgG)	38.4±56.2	18.6±8.4	28.4±28.2	17.6±6.9	12.7±0.99	24.5
Anti-B2-Glycoprotein I (IgG)	15.3±47.5	8.9±7.6	4.3±3.5	4.5±1.9	3.9±1.8	3.9±1.5
Anti-N-Methyl-D-Aspartate receptor*	28.80±12.41	----	39.26±10.79	21.41±2.44	19.63±1.93	25.16±0.45

NPSLE = neuropsychiatric systemic lupus erythematosus; Non-NPSLE = lupus patients without current or past NP manifestations; SLE-surgical = lupus patients who underwent elective surgery; SLE-septic meningitis.

A The NMDAR antibody was measure in 34 central NP SLE patients, 5 peripheral NP SLE patients, 49 non-NPSLE patients, 12 SLE-surgical patients, 5 SLE septic-meningitis patients, and 25 non-autoimmune patients.

B P<0.001 versus Central NPSLE

C P = 0.006 versus Central NPSLE

D P = 0.03 versus Central NPSLE

E Only patients who scored positive at baseline for the specific antibody were considered,

### Autoantibodies in serum at baseline and 6 months later

Six-months after the hospitalization, the NP manifestations were considered clinically quiescent in both NPSLE groups, and disease activity had decreased significantly in the non-NPSLE patients (Table 1).

A decrease in the levels of anti-dsDNA, anti-ribosomal P, anti-cardiolipin, anti-β_2_ glycoprotein-I, and anti-NMDAR antibodies was observed at six months in patients with central NPSLE who tested positive at baseline. This decrease was significant only for the last two autoantibodies. Among the patients with peripheral NPSLE who tested positive at baseline, the levels of anti-dsDNA and anti-ribosomal-P antibodies showed a non-significant decrease, while anticardiolipin and anti-β_2_ glycoprotein-I antibodies remained unchanged. In non-NPSLE patients who tested positive at baseline, a significant decrease in the levels of anti-dsDNA and anti-cardiolipin antibodies was observed; the levels of all the other autoantibodies remained unchanged (Table 3).

**Table 3:** Prevalence and levels of autoantibodies in serum of patients with central and peripheral NPSLE, and non-NPSLE at baseline and 6 months.

Autoantibodies positive, No. (%)	Central NPSLE Baseline (n = 34)	Central NPSLE 6 months (n = 34)	P (%)	Peripheral NPSLE Baseline (n = 5)	Peripheral NPSLE 6 months (n = 5)	P (%)	Non-NP-SLE baseline (n = 40)	Non-NP-SLE 6 months (n = 40)	P (%)
Antinuclear	28 (82)	25 (74)	0.38	4 (80)	4 (80)	1.0	40 (100)	31 (77)	0.001
Anti-ds DNA	27 (79)	20 (58)	0.07	5 (100)	3 (60)	0.22	30 (75)	28 (70)	0.62
Anti-ribosomal	21 (61)	16 (47)	0.22	4 (80)	5 (100)	1.0	13 (33)	13 (33)	1.0
Anti-cardiolipin, IgG	5 (15)	5 (15)	1.0	2 (40)	1 (20)	1.0	11 (28)	9 (23)	0.60
Anti-B2-Glycoprotein I, IgG	22 (65)	17 (50)	0.22	4 (80)	5 (100)	1.0	23 (58)	19 (48)	0.36
Anti-N-Methyl-D-Aspartate Receptor (NMDAR)^A^	8 (35)	7 (30)	0.75	0 (0)	2 (40)	0.44	12 (30)	10 (25)	0.62
**Autoantibodies levels** B									
Anti-ds DNA	238.8±519.9	41.6±76.7	0.06	163.9±292.5	37.9±38.7	0.22	533.04±722.2	162.1±399.5	0.008
Anti-ribosomal	96.0±157.8	46.5±87.3	0.08	106.9±188.7	25.8±19.8	1.0	69.3±98.7	63.5±105.2	0.10
Anti-cardiolipin, IgG	51.3±70.4	13.6±5.5	0.13	18.5±8.4	18.2±11.4	0.65	31.7±31.6	12.8±9.6	0.01
Anti-B2-Glycoprotein I, IgG	17.0±51.6	4.6±4.4	0.03	8.9±7.6	5.6±1.5	0.46	5.5±4.5	4.9±4.2	0.70
Anti-N-Methyl-D-Aspartate Receptor (NMDAR)	31.0±13.0	24.5±13.7	0.02	–	–	–	39.67±11.66	50.23±23.5	0.28

A The NMDAR antibody was measured in 23 central and 5 peripheral NPSLE, and in 40 non-NPSLE patients at baseline and 6 months.

B Only patients who scored positive at baseline for the specific antibody were considered, Central NPSLE/Peripheral NPSLE/non-NPSLE: anti-dsDNA 27/5/30, anti-ribosomal P 21/4/13, anticardiolipin IgG 5/2/11, anti-β_2_-glycoprotein-I 22/4/23, anti-NMDAR 8/0/12.

### Autoantibodies in CSF

In CSF, anti-nuclear, anti-dsDNA, anti-cardiolipin, and anti-β_2_ glycoprotein antibodies did not show a distinctive pattern across the SLE groups. In general, patients with septic meningitis had the highest prevalence for almost all the antibodies (Table 4).

**Table 4:** Prevalence and levels of autoantibodies in cerebrospinal fluid from patients with NPSLE and controls.

Autoantibodies Positive, no. (%)	Central NPSLE (35)	Peripheral NPSLE (n = 5)	SLE-Surgical (n = 16)	SLE-septic meningitis (n = 5)	Non-autoimmune (n = 17)
Antinuclear	20 (57)	5 (100)	7 (44)	5 (100)	0 (0) 1
Anti-ds DNA	27 (77)	3 (60)	10 (63)	4 (80)	0 (0)^2^
Anti-ribosomal P	16 (46)	2 (40)	5 (31)	5 (100)^3^	1 (6)^3^
Anti-cardiolipin, IgG	4 (11)	0 (0)	2 (13)	3 (60)^4^	0 (0) 4
Anti-B2-Glycoprotein I, IgG	0 (0)	0 (0)	9 (56)^5^	0 (0)	0 (0)
Anti-N-Methyl-D-Aspartate receptor (NMDAR)^7^	14 (41)	0 (0)	1 (8)^6^	5 (100)^6^	1 (4)^6^
**Autoantibodies levels** 8					
Anti-ds DNA	1007.5±2924.2	237.4±390.1	24.3±19.6	23.8±18.3	—
Anti-ribosomal P	542.7±1649.3	30.9±14.4	148.8±196.7	632.1±962.9	10.5
Anti-cardiolipin, IgG	10.7±2.7	—	17.6±6.9	19.5±9.8	—
Anti-B2-Glycoprotein I, IgG	—	—	4.5±1.9	—	—
Anti-N-Methyl-D-Aspartate receptor (NMDAR)^7^	80.8±27.9	—	40.3	89.7±49.8	63.0

NPSLE = neuropsychiatric systemic lupus erythematosus; SLE-surgical = lupus patients who underwent elective surgery; SLE-septic meningitis = lupus patients with septic meningitis, Non-autoimmune = patients without autoimmune diseases.

1 P<0.004 versus all SLE groups

2 P<0.007 versus all SLE groups

3 P<0.005 versus Central NPSLE and SLE-septic meningitis

4 P = 0.006 versus SLE-septic meningitis

5 P<0.05 versus all the other groups

6 P<0.02 versus Central NPSLE

7 The NMDAR antibody was measure in 34 SLE-CNS patients, 5 SLE-PNS, 12 SLE-surgical, 5 SLE-septic meningitis, and 25 Non-autoimmune patients.

8 Autoantibodies levels represent the mean (±SD) among the patients in whom they were positive. The number of patients in each group for a particular antibody corresponds to the prevalences displayed in the upper panel.

Anti-ribosomal P antibodies did not demonstrate a characteristic distribution across the SLE groups either, except in patients with septic meningitis where a significantly higher prevalence than in patients with central NPSLE was detected. Although the levels of anti-ribosomal P antibodies tended to be higher in central NPSLE patients than among the peripheral NPSLE and the surgical groups, the highest levels were found in patients with septic meningitis.

Anti-NMDAR antibodies were the sole antibodies showing a distinctive distribution in SLE. Except in one patient from the SLE surgical and one from the non-autoimmune group, they were found mostly in patients from the central NPSLE and septic meningitis groups, and no patient with peripheral NPSLE tested positive. The levels of anti-NMDAR antibodies were higher in patients with central NPSLE than in the other SLE groups, except in patients with septic meningitis in whom the highest levels were detected. (Table 4).

### Autoantibodies in CSF at baseline and 6 months later

Six months after the onset of the central and peripheral NP manifestations, the prevalence of most antibodies studied remained similar to the determination at baseline (Table 5).

**Table 5:** Prevalence and levels of autoantibodies in cerebrospinal fluid of patients with central and peripheral NPSLE at baseline and 6 months^A^

Autoantibodies positive, no. (%)	Central NPSLE Baseline (n = 25)	Central NPSLE 6 months (n = 25)	P (%)	Peripheral NPSLE Baseline (n = 5)	Peripheral NPSLE 6 months ( n = 5)	P (%)
Antinuclear	15 (60)	11 (44)	0.26	5 (100)	4 (80)	1.0
Anti-ds DNA	17 (68)	14 (53)	0.61	3 (60)	2 (40)	1.0
Anti-ribosomal	10 (40)	9 (36)	0.95	2 (40)	2 (40)	1.0
Anti-cardiolipin (IgG)	4 (16)	0 (0)	0.11	0 (0)	0 (0)	–
Anti-B2-Glycoprotein I (IgG)	0 (0)	0 (0)	–	0 (0)	0 (0)	–
Anti-N-Methyl-D-Aspartate Receptor (NMDAR)	9 (39)	5 (22)	0.34	0 (0)	2 (40)	0.44
**Autoantibodies levels** B						
Anti-ds DNA	463.3±1815.1	17.2±17.3	0.03	237.4±390.1	10.8±3.7	0.10
Anti-ribosomal	851.4±2061.6	25.1±21.6	0.01	30.9±14.4	12.5±3.1	0.17
Anti-cardiolipin (IgG)	10.7±2.7	4.0±0.5	0.06	–	–	–
Anti-N-Methyl-D-Aspartate Receptor (NMDAR)	82.9±39.9	76.3±93.1	0.26	–	–	–

A The baseline evaluation was at the time of hospitalization. The NMDAR antibody was measured in 23 central and 5 peripheral NPSLE patients at baseline and 6 months.

B Only patients who scored positive at baseline for the specific antibody were considered: Central NPSLE/Peripheral NPSLE anti-dsDNA 17/3, anti-ribosomal P 10/2, anticardiolipin IgG 4/0, anti-NMDAR 9/0.

Among the patients with central NPSLE who tested positive at baseline, a decrease in the levels of anti-dsDNA, anti-ribosomal P, and anti-NMDAR antibodies was observed in 14 out of 17, 9 out of 10, and 7 out of 9 patients, respectively. A significant decrease in the levels of anti-dsDNA and anti-ribosomal P antibodies was observed at six months.

Although the levels of anti-NMDAR antibodies seemed to remain unchanged, this is due to one patient in whom at six months, the levels increased 3.6 fold in comparison to baseline. When this patient is excluded from the analysis, a significant decrease is also observed (82.3±33.0 vs. 46.4±26.7, P = 0.04).

Among the patients with peripheral NPSLE, a non-significant decrease in the levels of anti-dsDNA and anti-ribosomal P antibodies was also detected (Table 5).

## Discussion

The detection of autoantibodies in serum or CSF in NPSLE patients has been studied for decades [23]. Unfortunately, the association between antibodies and nervous system involvement in SLE remains inconclusive. Moreover, the mechanisms whereby serum and CSF antibodies reactive with neural tissue are elicited remain an enigma [16].

In this study, we found a different behaviour of autoantibodies in serum and CSF at the onset of NPSLE and six months later, when clinically NP manifestations seemed to be quiescent.

In serum, anti-ribosomal P and anti-NMDAR antibodies were detected in most SLE groups, and did not differentiate among groups. Other antibodies implicated in thrombogenesis also did not show clear differences across the SLE groups, and the levels of all antibodies did not differ among them. Six-months after the hospitalization, the prevalence of almost all the autoantibodies remained unmodified and the decrease in the levels of some of them appears to reflect an overall decline in lupus activity.

In CSF, anti-nuclear, anti-dsDNA, anti-cardiolipin, anti-β_2_ glycoprotein-I, and anti-ribosomal P antibodies did not show a distinctive pattern across the SLE groups. In contrast, anti-NMDAR antibodies were found mostly in patients with central NPSLE and in patients with septic meningitis, and only in one out of 16, and one out of 17 patients from the surgical SLE and the non-autoimmune disease groups, respectively. In addition, the levels of anti-NMDAR antibodies were significantly higher in patients with central NPSLE than in the peripheral NPSLE and the surgical SLE groups, but not in patients with septic meningitis. Most patients with central, but not peripheral, NPSLE who tested positive for anti-dsDNA, anti-ribosomal P, and anti-NMDAR antibodies at baseline, had a decrease in the levels of these antibodies at six months.

The contrasting scenario found in CSF and serum exposes the unreliability of assessing neurological involvement in SLE patients by measuring serum autoantibodies. CSF is employed as an indirect assessment of what could be occurring in the CNS, since direct access and collection of brain tissue is rarely justified. The presence of antibodies in the CSF has a twofold explanation: i) *in situ* production in the CNS, which has been related to certain diseases such as multiple sclerosis [24], and ii) a breach in the blood-brain barrier (BBB), which would allow antibodies access to a normally restricted compartment [25]. This may explain the presence and the high levels of all sorts of autoantibodies found in SLE patients with septic meningitis, since in this condition there is a clear disruption of the BBB.

Measurement of antibodies in the CSF revealed anti-NMDAR antibody as an important potential protagonist that was virtually unnoticed in serum, and this specificity showed a radically different behaviour from the other antibodies studied. It was the only antibody that differentiated patients with central from those with peripheral NPSLE and from the surgical SLE group. Moreover, high levels of anti-NMDAR antibody were significantly associated with central NPSLE.

It is worth noting, however, that in the same SLE patients we detected the presence of all the antibodies tested, except for anti-NMDAR antibody. This implies that these patients have had, during the course of their illness, a breach in their BBB that allows access of antibodies to the CNS. Although infection, stress, hypertension, or exposure to nicotine may disrupt the BBB [26]–[29], it is possible that in lupus flares, the immunological and inflammatory alterations that occur might allow serum antibodies into the CSF. Another plausible explanation would be that the breach of the BBB could be a sporadic but recurring phenomenon in SLE patients. Indeed, pathologic studies of patients with NPSLE who died have shown that true vasculitis of small vessels, the hallmark of lupus pathology, is generally missing [30]. There are widely scattered microinfarcts and a non-inflammatory vasculopathy, characterized by intimal proliferation and perivascular gliosis [31]. This bland vasculopathy does not generally correlate with the clinical findings, and it may be present in patients without NP involvement. Thus, antibodies may gain entry into the CNS through an alteration of the BBB, perhaps resulting from damage visualised as bland vasculopathy.

When clinically NP manifestations had been resolved, we did not observe a decrease in CSF in the prevalence of the autoantibodies tested in patients with central and peripheral NPSLE. Nevertheless, among the patients with central NPSLE who tested positive at baseline, a decrease in the levels of anti-dsDNA, anti-ribosomal P, and anti-NMDAR antibodies was observed. This observation may suggest that their presence could be conditioning a sort of damage that we were unable to detect clinically or radiologically.

Recently, we reported the participation of IL-6 and chemokines in these patients and showed that the levels of practically all the molecules that had originally shown high concentrations decreased significantly, although not as far as the levels found in the patients without autoimmune diseases [32]. Rather, they reached a range of values that could be defined as the basal level in SLE, since they were similar to the levels found in the non-NPSLE patients. The same seems to occur with autoantibodies. This is of the highest importance, because SLE patients show long term neurological damage, which manifests itself as a decrease in brain volume and a worsening of cognitive function [33], [34]. Although this worsening is greater in patients with history of NPSLE, it is also observed in patients without previous NP manifestations [35]. One must emphasize that the anti-NMDAR antibody has been categorized as a mediator for cognitive impairment [17]. There is no recognized aetiology of these alterations; it is possible, however, that this slight, but persistent increase in chemokines and antibodies levels in the CSF of SLE patients elicits this damage by maintaining a chronic subclinical inflammation or progressive neuronal loss.

### Limitations

In order to define the borders of our results, we must highlight some potential limitations. 1) Although a relatively large number of patients with NP manifestations were included and we did detect a correlation between CNS disease and specific antibodies, we were unable to correlate specific antibodies with specific manifestations, since the study was not adequately powered for them. Therefore, our results should be interpreted with caution since NPSLE manifestations are likely to be due to heterogeneous mechanisms [36]. 2) Furthermore, a limited number of patients with peripheral NP manifestations were included, which could raise concerns about the generalization of our results. The pathogenic mechanism of these manifestations, however, is likely to be different from that of central manifestations, and we therefore consider that the results obtained, illustrate the behaviour of the antibodies studied in the central and peripheral NP manifestations. 3) We included patients with NP manifestations attributable to SLE according to the ACR nomenclature, rejecting patients with exclusion factors for the attribution of the NP manifestations [20], and patients with minor events that have a comparable frequency in the normal population [21]. Nevertheless, in 48 percent of our patients, there were concurrent non-SLE factors, which might have contributed to the development of symptoms. Since this is a complex issue, some misclassification in the attribution may be present. 4) Our results apply to patients with acute NP manifestations who needed to be hospitalized for their diagnosis and/or treatment. We did not study non-hospitalized patients, or patients with chronic serious manifestations, e.g. depression, seizures or cognitive dysfunction. 5) We did not carry out an in-depth screening for NP manifestations among the patients without history for them, therefore although none of them had any of the acute and severe manifestations included among the patients with central and peripheral NPSLE, we cannot exclude that they had mild or subclinical NP manifestations. 6) Although at six months, the NPSLE patients were clinically in remission, we cannot exclude ongoing, subclinical NP activity which might associate with the presence and levels of the antibodies studied in CSF.

Our study also has strengths that need to be emphasized. 1) This is the first study to assess the levels of several antibodies simultaneously in serum and in CSF, in various groups of patients with SLE and in subjects without autoimmune disease. Thus, several groups of negative controls were assembled as well as SLE patients with infectious meningitis in whom an inflammatory global process in the meninges, not due to lupus activity, was present. This positive control group allows us to determine the specificity of the antibodies studied as markers of NP activity due to lupus. 2) The paired assessment in serum and CSF of autoantibodies during the acute episode and six months later provides unique information about the sensitivity of the levels of autoantibodies to change in the status of NP manifestations. 3) All patients were assessed prospectively following a standardized evaluation, and the antibody determination was blinded to the diagnosis and the study group. Furthermore, all patient sera and CSF was evaluated simultaneously to avoid intra-assay variabililty.

Overall, our results provide answers and more questions in the complex challenge that represents CNS involvement in SLE.

1. We clearly show a lack of correlation of serum antibodies to NPSLE manifestations.
2. We also demonstrate that when studying the pathogenesis of NPSLE, it will be important to subdivide CNS and PNS manifestations.
3. We also show that longitudinal studies may help reveal the role of antibodies in acute manifestations of NPSLE.

Several questions remain, which should be answered with future research:

- Do anti-ribosomal P and anti-NMDAR antibodies contribute to CNS damage in patients with SLE and infectious meningitis, with the clinical manifestations of meningitis masking the symptoms of NPSLE?
- Do the selective expression of NMDARs in the CNS account for the exquisite tropism towards CNS on the part of anti-NMDAR antibody and not of the other antibodies studied?
- Why does the anti-NMDAR antibody persist in CSF for such a long time at a high level? Alternatively, why does this antibody cross the BBB persistently and in a selective manner in central NPSLE patients? Does the continued presence of this antibody explain the insidious nature of the cognitive impairment observed in SLE patients?

In conclusion, our results do not support the quantification in serum of the autoantibodies tested as a diagnostic tool; however, we believe that they play an important role in the pathogenesis of NPSLE, particularly the anti-NMDAR antibody in central nervous system involvement. The concomitant measurement of other potentially involved molecules such as cytokines is needed to delineate the molecular basis for each NP manifestation, focusing on CSF samples. The damage to the BBB both in NPSLE and non-NPSLE patients has been underestimated [17], [25]. It is therefore essential to aim our efforts at trying to ascertain the mechanisms responsible for altering the BBB integrity, and to reduce the process leading to the CNS involvement in SLE.
